# Merkel Cell Carcinoma of the Thigh Presenting as a Hemorrhagic Mass: A Rare Case Report and Literature Review

**DOI:** 10.3390/curroncol33040204

**Published:** 2026-04-01

**Authors:** Hüseyin Emre Tepedelenlioğlu, Özlem Orhan, Şefik Murat Arıkan, Güldal Esendağlı

**Affiliations:** 1 Department of Orthopedics and Traumatology, Ankara Etlik City Hospital, Ankara 06170, Turkey; 2 Department of Orthopedics and Traumatology, Abdurrahman Yurtaslan Oncology Training and Research Hospital, Ankara 06200, Turkey; droorhan@gmail.com; 3 Department of Orthopedics and Traumatology, Faculty of Medicine, Gazi University, Ankara 06560, Turkey; smuratarikan@gazi.edu.tr; 4 Department of Pathology, Faculty of Medicine, Gazi University, Ankara 06560, Turkey; guldal@gazi.edu.tr

**Keywords:** Merkel cell carcinoma, thigh, lower extremity, inguinal lymph node, sentinel lymph node biopsy, adjuvant radiotherapy, Merkel cell polyomavirus

## Abstract

Background: Merkel cell carcinoma (MCC) is a rare, aggressive primary cutaneous neuroendocrine carcinoma with a marked propensity for early regional lymph node metastasis. Although MCC most often arises on sun-exposed head and neck skin in older adults, tumors of the lower extremity are uncommon and may be mistaken for benign hemorrhagic lesions. Case presentation: A 54-year-old woman developed a rapidly enlarging, hemorrhagic mass in the left suprapatellar thigh. Magnetic resonance imaging demonstrated an extracompartmental subcutaneous soft-tissue mass without quadriceps muscle invasion. Wide local excision including the quadriceps fascia was performed. Histopathologic examination showed a dermal/subcutaneous small blue round cell neoplasm with brisk mitotic activity. Immunohistochemistry demonstrated diffuse cytoplasmic synaptophysin positivity, paranuclear dot-like CK20 reactivity, chromogranin A positivity, and negative MCPyV staining; TTF-1, S100, melan-A, HMB-45, and hematolymphoid markers were negative. Staging positron emission tomography/computed tomography identified ipsilateral inguinal nodal involvement. Therapeutic inguinal lymph node dissection revealed metastatic MCC in one of four lymph nodes without extranodal extension. The final stage was pT3 pN1b cM0 (AJCC 8th edition), corresponding to stage IIIB disease. Adjuvant radiotherapy (57 Gy in 20 fractions) was delivered to the primary bed and ipsilateral inguinal basin. The patient remains disease-free at 5-year follow-up. Conclusions: Lower-extremity MCC can mimic hemorrhagic or post-traumatic lesions, contributing to diagnostic delay. Persistent or rapidly enlarging “hematoma-like” lesions warrant early biopsy, and timely pathologic nodal staging is essential. Multimodal management can achieve durable control even in node-positive disease

## Simple Summary

Merkel cell carcinoma, a rare and aggressive skin cancer, tends to metastasize early to regional lymph nodes. This cancer tends to occur on sun-exposed areas such as the head and neck, and thigh lesions may be overlooked or mistaken for benign bleeding or trauma-related masses, especially when they occur on sun-protected areas such as the thigh. This article reports a case of a woman presenting with a rapidly growing, bleeding mass above the knee, resembling a hematoma, and diagnosed with Merkel cell carcinoma following tissue evaluation, with further evaluation showing metastasis to a groin lymph node. The cancer was controlled with a combination of surgery and radiation, and there has been no evidence of recurrence in five years. This case highlights the importance of promptly investigating any bleeding mass, especially if it has a tendency to persist or enlarge rapidly, to prevent delayed diagnosis and improve patient outcomes.

## 1. Introduction

Merkel cell carcinoma (MCC) is a rare and highly aggressive primary cutaneous neuroendocrine carcinoma characterized by rapid growth, early regional lymph node spread, and substantial disease-specific mortality [1]. In the United States, the incidence reached approximately 0.7 cases per 100,000 person-years in 2013, corresponding to about 2488 new cases annually, and demographic modeling projected that the annual case burden would exceed 3000 cases by 2025, with contemporary analyses suggesting stabilization in more recent years and improving survival in the modern multidisciplinary and immunotherapy era [1,2]. More recent epidemiologic work suggests that most MCC cases in the United States are attributable to either ultraviolet radiation (UVR) exposure or Merkel cell polyomavirus (MCPyV), whereas only a small fraction is attributable to major immunosuppressive conditions alone [3]. MCC arises most commonly on chronically sun-exposed skin in older, fair-skinned individuals, and the AEIOU heuristic (Asymptomatic, Expanding rapidly, Immune suppression, Older than 50 years, UV-exposed/fair skin) remains a useful clinical summary [4].

Two major pathogenetic pathways are recognized. Virus-positive tumors are driven by MCPyV integration and viral T-antigen expression, whereas virus-negative tumors are typically characterized by UV-signature mutagenesis, a high tumor mutational burden, and recurrent genomic instability [5,6]. Although MCC classically involves the head and neck, primary tumors may arise on the trunk and extremities, where they can mimic benign or post-traumatic lesions and delay diagnosis. We report a large suprapatellar thigh MCC presenting as a hemorrhagic mass and discuss the diagnostic, immunohistochemical, and molecular implications of an MCPyV-negative tumor in this unusual location.

## 2. Case Presentation

**Figure 1:**
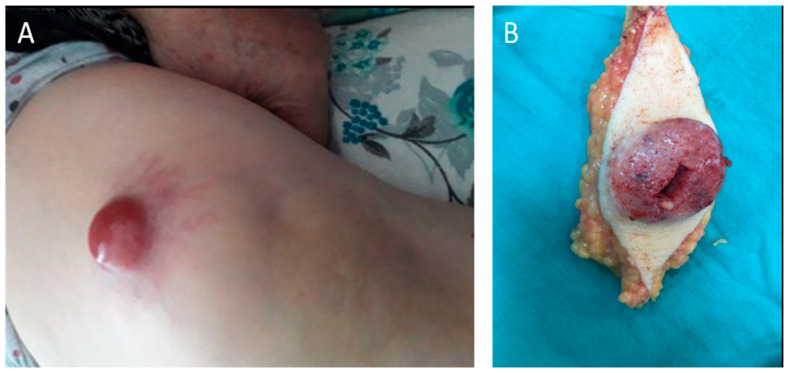
Clinical and gross findings. (**A**) Exophytic hemorrhagic lesion in the left suprapatellar thigh at presentation. (**B**) Gross specimen following wide local excision including underlying fascia.

A 54-year-old woman presented with a skin lesion in the left suprapatellar region that had been present for approximately 2 months. The lesion had enlarged markedly over the preceding 2 weeks and began bleeding (Figure 1A). Physical examination demonstrated an exophytic, hemorrhagic mass without systemic symptoms.

**Figure 2:**
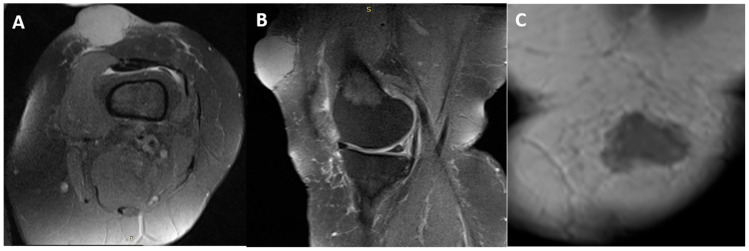
T2 post-contrast magnetic resonance imaging of the left distal thigh demonstrating an extracompartmental subcutaneous mass without quadriceps muscle invasion (representative (**A**) axial, (**B**) sagittal and (**C**) coronal images).

Magnetic resonance imaging revealed an extracompartmental soft-tissue mass with irregular margins in the subcutaneous tissue of the distal thigh, without necrosis and without invasion of the quadriceps musculature (Figure 2). Because malignancy was suspected, the lesion was treated surgically with wide local excision including the underlying quadriceps fascia; intraoperative frozen section supported malignancy (Figure 1B).

**Figure 3:**
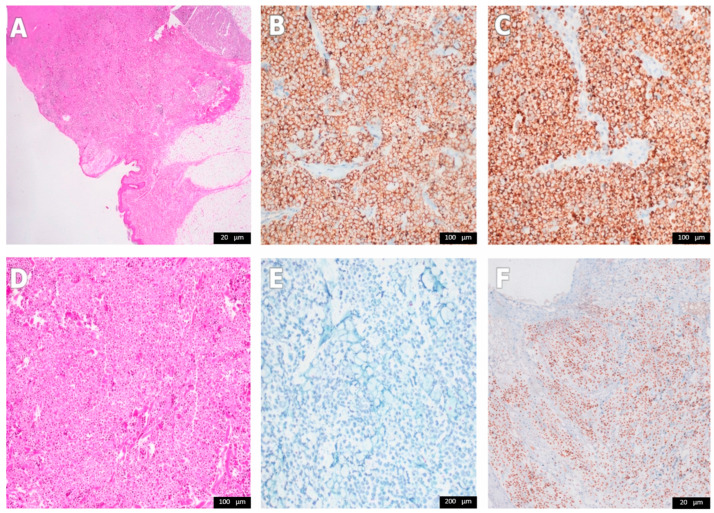
Histopathology and immunohistochemistry. (**A**) Low-power hematoxylin and eosin (H&E) view of a dermal/subcutaneous malignant neoplasm. (**B**) Synaptophysin immunostain showing diffuse cytoplasmic positivity. (**C**) CK20 immunostain showing classic paranuclear dot-like reactivity. (**D**) Higher-power H&E section showing sheets and nests of small basophilic tumor cells. (**E**) MCPyV immunostain is negative. (**F**) INSM1 immunostain showing diffuse nuclear positivity in tumor cells.

Gross examination showed a gray-white tumor measuring 9.0 × 4.5 × 3.5 cm involving the skin and subcutaneous tissue. Microscopy demonstrated a dermal/subcutaneous infiltrate of small basophilic tumor cells with a high mitotic rate (30 mitoses/mm^2^), scant cytoplasm, and focal perineural invasion; lymphovascular invasion was not identified (Figure 3A,D,F). Immunohistochemistry showed diffuse cytoplasmic synaptophysin positivity and paranuclear dot-like CK20 reactivity, together with chromogranin A positivity, supporting MCC (Figure 3B,C). MCPyV immunostaining was negative (Figure 3E). A positivity and diffuse nuclear INSM1 positivity were found, supporting MCC (Figure 3B,C,F). The tumor was also negative for TTF-1, S100, melan-A, HMB-45, CD45, CD3, CD20, vimentin, cytokeratin MNF116, CD38, CD138, CD23, and myeloperoxidase. Ki-67 proliferative activity was approximately 80–85%. Surgical margins were negative, with a minimum margin of 1.8 cm.

Staging positron emission tomography/computed tomography demonstrated ipsilateral inguinal nodal involvement without distant metastasis. Therapeutic inguinal lymph node dissection removed four nodes, of which one contained metastatic MCC without extranodal extension. The final stage was pT3 pN1b cM0 (AJCC 8th edition [7]), corresponding to stage IIIB disease. Adjuvant external-beam radiotherapy (57 Gy in 20 fractions) was delivered to the primary bed and ipsilateral inguinal basin. The patient remains disease-free at 5-year follow-up.

## 3. Results and Discussion

MCC is a rare cutaneous neuroendocrine carcinoma characterized by rapid growth, but its clinical significance is disproportionate to its rarity because of its aggressive biology and high frequency of nodal dissemination. The present case broadens the clinicopathologic spectrum of lower-extremity MCC by showing that a large suprapatellar lesion may present as a hemorrhagic, hematoma-like mass and initially enter the differential diagnosis of soft-tissue tumor or post-traumatic lesion. This pattern matters clinically because a delayed biopsy may postpone appropriate staging and nodal management.

The histopathologic differential diagnosis of MCC includes metastatic small cell neuroendocrine carcinoma, especially small cell lung carcinoma, as well as melanoma, lymphoma/leukemia, Ewing-family tumors, and other cutaneous basaloid malignancies. In routine practice, the diagnosis rests on integration of morphology with an immunophenotypic panel rather than reliance on a single marker. The present tumor showed the classic MCC pattern of CK20 positivity with a dot-like paranuclear distribution together with neuroendocrine marker expression and TTF-1 negativity. This constellation strongly supports MCC over small cell lung carcinoma, which is usually CK20 negative and frequently TTF-1 positive [7,8,9,10]. The CK20 staining pattern is diagnostically important: in MCC, the characteristic perinuclear globular or dot-like reactivity reflects aggregated intermediate filaments and remains one of the most useful practical clues in distinguishing MCC from other high-grade neuroendocrine carcinomas [8,9].

Additional markers may be useful in difficult or limited specimens. INSM1 is a highly sensitive nuclear marker of neuroendocrine differentiation and has shown more homogeneous staining than conventional neuroendocrine markers in MCC, although it does not by itself distinguish MCC from metastatic extracutaneous neuroendocrine carcinomas [10,11,12,13]. In our case, INSM1 immunostaining was subsequently performed and showed diffuse nuclear positivity (Figure 3F), providing additional support for the diagnosis. More recently, POU4F3 has emerged as a highly sensitive and relatively specific nuclear marker for MCC and may be particularly valuable in small biopsies and sentinel lymph node specimens when CK20 is negative or equivocal [14]. POU4F3 was not available for this case; therefore, the diagnosis rests on morphology, CK20 dot-like reactivity, neuroendocrine marker expression, negative TTF-1, and positive INSM1.

The negative MCPyV immunostain in our case is noteworthy because it raises the possibility of a virus-negative, UV-associated subtype. Biologically, MCC can be divided into virus-positive and virus-negative tumors, and these groups differ in antigenic drivers, genomic architecture, and likely therapeutic vulnerabilities. Virus-positive tumors are driven by integration of MCPyV with continued expression of oncogenic viral large T and small T antigens and usually harbor a relatively low mutational burden [5,6]. By contrast, virus-negative tumors are typically characterized by UV-signature DNA damage, substantially higher tumor mutational burden, recurrent RB1 and TP53 alterations, and greater copy-number complexity [15,16]. Starrett et al. further showed that high-confidence viral detection improves classification of MCC and that virus-negative tumors may also be associated with immunosuppression and inferior overall survival in some cohorts [15].

The biologic implications of an MCPyV-negative phenotype extend beyond etiology. UV-associated tumors are likely to generate abundant neoantigens because of their high mutational burden, whereas virus-positive tumors may instead be immunogenic through viral oncoproteins [6,15]. This distinction is relevant to tumor–immune interactions and may partly explain why both biologic classes can respond to immune checkpoint blockade despite fundamentally different antigenic landscapes. Additional work has also shown that epigenetic events contribute to MCC progression and immune escape. In multicenter studies, higher PDCD1 promoter methylation and higher hTERT intron 4–5 methylation were associated with adverse clinicopathologic features and worse overall survival, underscoring that MCC biology is shaped not only by viral status and UV mutagenesis but also by epigenetic regulation [17,18]. Because only MCPyV immunohistochemistry was available in this case, our classification is appropriately limited to an MCPyV-negative immunophenotype rather than genomically confirmed virus-negative disease.

Accurate staging is central to MCC management. AJCC 8th edition staging emphasizes primary tumor size and nodal status, and occult nodal metastases are common even in clinically localized disease [19]. Accordingly, current guideline frameworks recommend careful pathologic nodal evaluation, multidisciplinary decision-making, and risk-adapted use of radiotherapy [20,21]. For recurrent locally advanced or metastatic disease, immune checkpoint inhibitors targeting the PD-1/PD-L1 axis have become the preferred systemic approach in most contemporary algorithms [1,20,21,22,23]. In our patient, wide excision, therapeutic inguinal lymph node dissection, and adjuvant radiotherapy achieved durable local–regional control and prolonged disease-free survival.

To contextualize this presentation, we reviewed published English-language reports of primary thigh and lower-extremity MCCs. As summarized in Table 1, these cases repeatedly emphasize rapid enlargement, diagnostic uncertainty, and frequent nodal involvement at diagnosis or during follow-up [23,24,25,26,27,28]. The present case adds to this literature by documenting a large suprapatellar hemorrhagic mass with pathologically confirmed inguinal nodal metastasis and durable control after multimodal treatment. The case also illustrates how a seemingly hematomatous lesion can conceal a biologically aggressive cutaneous neuroendocrine carcinoma.

## 4. Conclusions

This case emphasizes that MCC of the thigh may present as a hemorrhagic, hematoma-like mass and may initially fall outside usual dermatologic diagnostic pathways. Early biopsy of persistent or rapidly enlarging hemorrhagic lesions, careful immunohistochemical work-up that includes attention to dot-like CK20 reactivity, and timely nodal staging are essential. Discussion of the MCPyV-negative/UV-associated subtype is also important because it frames the biologic heterogeneity of MCC and its potential molecular and therapeutic implications.

## Figures and Tables

**Table 1:** Reported primary Merkel cell carcinoma cases of the thigh/lower extremity and key clinical features, treatment, and outcomes (including the present case).

First Author (Year)	Age/Sex	Primary Site	Presentation/Diagnostic Pitfall	Tumor Size (cm)	Nodal/Stage Details	Stage	Treatment	Outcome/Follow-Up
Jiang (2019) [24]	86/M	Right thigh	Red nodule; initially interpreted as “subcutaneous small cell cancer” on outside pathology	2.5 × 2.0 × 1.2	T2N1M0; inguinal nodal metastasis on CT (~2.2 cm), no distant mets	T2N1M0	Excision, then anti-angiogenic therapy (endostar + apatinib)	Partial response at 2 months; PFS 6.5 months; OS 13 months
Zhang (2022) [25]	90/F	Right medial thigh (pretibial/near knee)	Dark red pruritic nodule; MRI showed hypervascular subcutaneous lesion	~3 × 3	Inguinal nodes palpable clinically; path nodal evaluation not described	Not reported	Wide local excision with 2 cm margin; patient declined adjuvant radiotherapy	No recurrence at 30 months
Limardo (2017) [26]	62/M	Right thigh (appeared after nodal presentation)	Atypical sequence: initial “inguinal node” mass (occult primary), later violet thigh skin nodule interpreted as primary	4.5 × 3.5 × 24	Initial inguinal disease; later thigh primary suspected; CK20/CD56/chromogranin/synaptophysin positive; TTF-1 negative	Not reported	Inguinal lymphadenectomy, radiotherapy (5800 cGy), later thigh resection with flap reconstruction	Disease-free at 24 months
Guadagni (2019) [27]	73/F	Left calcaneal region	Recurrent/in-transit limb nodules after initial surgery; treatment constrained by comorbidity (active hepatitis C, neutropenia)	1.8 × 0.7; multiple new nodules	Initial SLNB negative; later developed ipsilateral inguinal node involvement during subsequent recurrences	Not reported	Repeated excisions plus isolated pelvic/limb perfusion with melphalan	Alive and disease-free at 56 months
Howell (2018) [28]	68/M	Left buttock (proximal lower extremity region)	Misread as gluteal abscess; foul-smelling drainage; CT showed large lesion + bulky inguinal LAD	Lesion on exam ~10 × 10; CT ~9 × 10.8 × 4.2 cm	Reported as stage 3B; inguinal LAD on imaging	Stage IIIB	Debridement and incisional biopsy followed by definitive excision, radiotherapy, and chemotherapy	Outcome not fully detailed
Al Diab (2013) [29]	38/M	Left thigh (near buttock)	Painless swelling; highlights young age/atypical demographic	Not reported	Not reported	(reported)	Inguinal lymphadenectomy and adjuvant local radiotherapy	Outcome not fully detailed
Present case	54/F	Left suprapatellar thigh	Hemorrhagic/hematoma-like mass; managed initially as suspected soft-tissue tumor	9 × 4.5 × 3.5	pT3 pN1b cM0 (stage IIIB); PET-positive ipsilateral inguinal node; dissection 1/4 positive; no extranodal extension	Not reported	Wide local excision including fascia, therapeutic inguinal lymph node dissection, and adjuvant radiotherapy (57 Gy/20 fractions)	No recurrence at 5 years

Abbreviations: CT, computed tomography; LAD, lymphadenopathy; MRI, magnetic resonance imaging; OS, overall survival; PFS, progression-free survival; SLNB, sentinel lymph node biopsy.

## Data Availability

The original contributions presented in the study are included in the article; further inquiries can be directed to the corresponding author.
